# Durable stable disease and immune activation in metastatic uveal melanoma treated with tebentafusp: case report

**DOI:** 10.3389/fimmu.2026.1864115

**Published:** 2026-07-02

**Authors:** Parham Habibzadeh, Anushri Kulkarni, Drew Hurd, Amy Rose, Danielle Bednarz, Yana G. Najjar, John M. Kirkwood, Arivarasan Karunamurthy, Diwakar Davar

**Affiliations:** 1 Department of Medicine, University of Pittsburgh Medical Center, Pittsburgh, PA, United States; 2 University of Pittsburgh Medical Center (UPMC) Hillman Cancer Center, Pittsburgh, PA, United States; 3 Division of Malignant Hematology and Oncology, Department of Medicine, University of Pittsburgh, Pittsburgh, PA, United States; 4 Division of Dermatopathology, Department of Dermatology, University of Pittsburgh, Pittsburgh, PA, United States

**Keywords:** case report, choroidal melanoma, ImmTAC, immunotherapy, tebentafusp, uveal melanoma, T cell engager

## Abstract

Metastatic uveal melanoma (mUM) is a rare, aggressive malignancy with limited therapeutic options in the metastatic setting. Tebentafusp (IMCgp100) is an immune-mobilizing monoclonal T cell receptor against cancer (ImmTAC), targeting gp100 in HLA-A*0201+ patients. Tebentafusp has demonstrated an overall survival (OS) benefit in clinical trials, usually without marked radiographic tumor regression. We describe a mUM patient who was treated with tebentafusp for more than two years and achieved durable stable disease on imaging but passed away due to sudden cardiac death. Autopsy examination revealed extensive tumor necrosis and dense immune cell infiltration within metastatic liver lesions with minimally viable tumor, providing direct evidence of robust immune activation in the tumor microenvironment. This case highlights the potential for T cell bispecifics to induce profound anti-tumor effects that are not fully captured by conventional radiographic assessment, underscoring the need for integrated clinical, pathologic, and imaging evaluation in this setting.

## Introduction

Uveal melanoma is the most common primary intraocular malignancy in adults; however, metastatic uveal melanoma (mUM) remains highly lethal, with median survival typically under 12 months (1). Conventional systemic therapies, including chemotherapy and immune checkpoint inhibitors (ICIs), have shown limited activity in this population (2). Tebentafusp (IMCgp100) is an immune-mobilizing monoclonal T cell receptor against cancer (ImmTAC) that redirects T cells to gp100-expressing melanoma cells in HLA-A*0201–positive individuals, providing a selective immunotherapeutic strategy (3). In a pivotal phase 3 trial (IMCgp100-202), tebentafusp improved overall survival (OS) by 6 months compared to investigator’s choice therapy (dacarbazine, ipilimumab, or pembrolizumab) in previously untreated mUM (4, 5). Recent data suggests that tebentafusp use facilitates macrophage reprogramming, resulting in a more favorable tumor microenvironment in mUM (6).

## Case presentation

An adult patient with HLA-A*0201–positive mUM presented with a history of choroidal melanoma of the left eye, previously treated with plaque brachytherapy. Following a nine-year disease-free interval, routine surveillance ultrasound identified indeterminate hepatic lesions, confirmed on contrast-enhanced CT and liver biopsy. Baseline staging revealed multiple hepatic lesions (largest 2 cm) and scattered peritoneal/serosal metastases (<1.3 cm). Lactate dehydrogenase (LDH) was 212 U/L (ULN 176 U/L); other laboratory parameters were unremarkable.

The patient’s prior medical history was notable for hypertension, hyperlipidemia, coronary artery disease (CAD) treated with two-vessel coronary artery bypass grafting and mitral valve prolapse status post mitral valve repair. At the time of study enrollment, the patient had preserved performance status, and had no cardiac symptoms, including chest pain or dyspnea. Periodic ECG assessment was done throughout the trial as part of cardiac safety monitoring, with no untoward findings. The patient remained on aspirin, atorvastatin, and amlodipine while he was enrolled in the trial.

**Figure 1:**
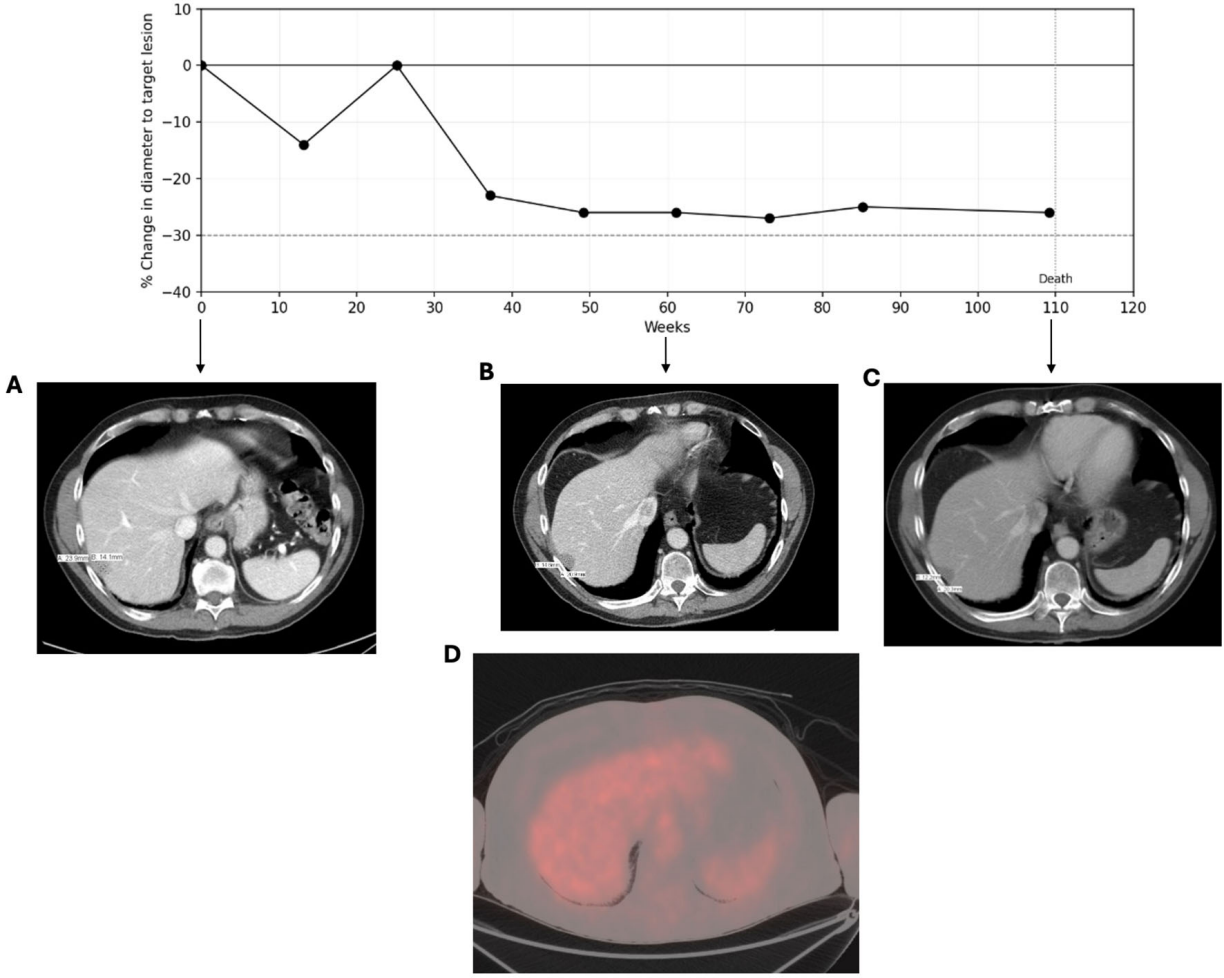
Pre- and on- treatment CT images of tebentafusp treated patient demonstrating representative hepatic lesions prior to **(A)**, 14 months after **(B)** initiation of tebentafusp therapy. CT images at timepoint in **(C)** were obtained 7 days prior to death. **(D)** Corresponding PET images from 22 months after the initiation of tebentafusp therapy.

**Figure 2:**
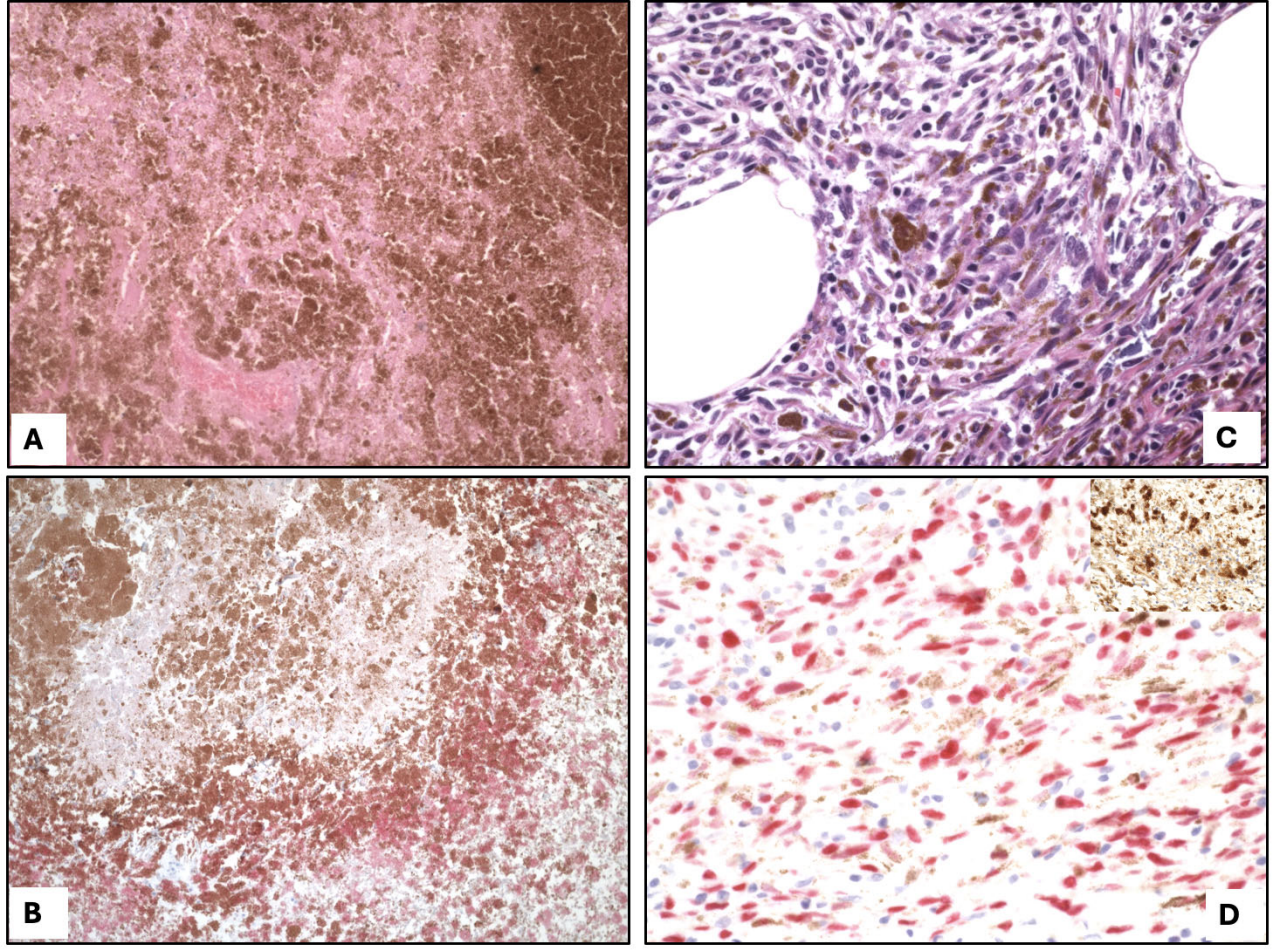
Histopathology examination using HE stained sections **(A)** show extensive tumor necrosis with melanin pigmentation and complete absence of viable tumor cells by melanA immunostain **(B)**. Adjacent sampled area show tumor bed with scattered melanoma cells **(C)** additionally highlighted by SOX-10 immunostain **(D)** and Melan A immunostain **(D)** inset with surrounding fibroblasts, lymphocytes, histiocytes and melanophages **(C)**.

The patient commenced tebentafusp monotherapy in August 2019. Over 37 cycles, the patient had regression of index hepatic lesions, although the degree of shrinkage did not exceed 30%, with consistent stable disease per RECIST v1.1 (**Figure 1**). Serial PET/CT imaging demonstrated non-FDG-avid lesions, indicating suppressed metabolic activity (**Figure 1D**). Four days following the completion of Cycle 37, the patient suffered sudden cardiac death and a rapid autopsy was arranged at the family’s behest. Post-mortem examination identified the immediate cause of death as acute myocardial infarction resulting from near-complete occlusion of the right coronary artery in the setting of multivessel coronary atherosclerosis. Histological evaluation of the myocardium revealed hyper-eosinophilic cardiomyocytes with contraction band necrosis, wavy fibers, and interstitial hemorrhage, consistent with acute myocardial ischemia. Background changes of chronic ischemic heart disease, including interstitial fibrosis and myocyte hypertrophy, were also present. Metastatic nodules within the liver were identified and subsequent histologic examination showed extensive tumor necrosis (**Figures 2A, B**) with melanin pigmentation and few viable tumor cells in the adjacent tumor bed stroma with fibrosis and lymphohistiocytic inflammation (**Figures 2C, D**).

## Discussion

This case provides autopsy-based evidence that tebentafusp therapy can induce profound immune-mediated remodeling of mUM, characterized by replacement of metastatic deposits with extensively necrotic tumor with melanin pigmentation and near complete absence of viable tumor along with adjacent histiocytic inflammation and prominent melanophagocytosis. These findings align with recent mechanistic data demonstrating that ImmTACs not only redirect T cells toward gp100-expressing tumor cells but also reprogram the myeloid compartment within the tumor microenvironment (6). Tebentafusp-driven T cell engagement leads to rapid cytokine and chemokine induction - particularly IFNγ, TNF, IL-6, and CXCL9/10 - which in turn recruit and activate macrophages, skewing them toward a pro-inflammatory, tumoricidal phenotype. Activated macrophages exhibit enhanced antigen presentation, phagocytic activity, and upregulation of HLA class II and co-stimulatory molecules, facilitating clearance of dying tumor cells and amplifying local antitumor immunity.

The extensive macrophage infiltration and melanophagocytosis observed at autopsy are consistent with such ImmTAC-mediated myeloid activation. The near complete absence of viable melanoma despite persistent radiographic disease highlights the discordance between imaging and tissue response that can arise when macrophage-driven clearance predominates over cytolytic tumor shrinkage. This phenomenon may contribute to the well-described dissociation between objective radiographic response and overall survival benefit with tebentafusp (4, 5). Immune-related skin changes such as vitiligo, also observed in this patient, are similarly consistent with systemic IFNγ-driven myeloid and T cell activation and melanin pigmentation clearance.

Cardiovascular events in patients receiving tebentafusp, particularly in those with pre-existing CAD, require careful interpretation. In the 3-year analysis of IMCgp100–202 and in a pooled safety analysis of 410 tebentafusp-treated patients (5, 7), tebentafusp-associated toxicities were predominantly cytokine-mediated and occurred mainly early during treatment. In the present case, autopsy demonstrated acute myocardial infarction due to near-complete right coronary artery occlusion in the setting of multivessel coronary atherosclerosis. The autopsy findings, together with the history of CAD, support an atherosclerotic mechanism for the fatal event. Although cytokine release, hypotension, and the subsequent hemodynamic stress could theoretically contribute to ischemia in susceptible patients, the available evidence does not establish a direct causal relationship between tebentafusp and myocardial ischemia in this patient.

Collectively, this case highlights the importance of integrating tissue-based analyses with clinical and radiographic assessments when evaluating ImmTAC therapies. Tebentafusp-mediated coordination of T cell–myeloid interactions may constitute a central mechanism of durable disease control in uveal melanoma, with macrophages functioning as active effectors of tumor clearance rather than passive responders. Further studies are warranted to identify biomarkers of myeloid activation, define the temporal dynamics of macrophage-mediated tumor elimination, and determine whether similar myeloid reprogramming is a generalizable feature of T cell receptor bispecific therapies across solid tumors. These observations also emphasize the need to better understand the dissociation between RECIST-based radiographic response and overall survival, and to assess the extent to which this phenomenon applies to TCR bispecifics beyond uveal melanoma.

The findings here also illustrate the limitations of relying exclusively on radiographic response criteria to assess tebentafusp activity. Although tissue biopsy can provide definitive evidence of treatment effect, repeated biopsy is invasive and impractical in routine care. Liquid biopsy approaches, particularly circulating tumor DNA, may provide a complementary and less invasive strategy for monitoring biologic response in metastatic uveal melanoma (8). Recent studies suggest that ctDNA detection and longitudinal ctDNA dynamics are associated with clinical outcomes in metastatic uveal melanoma, including in patients treated with tebentafusp (9, 10). Integration of serial imaging with blood-based biomarkers such as ctDNA may therefore better capture immune-mediated tumor clearance, particularly when residual radiographic lesions represent necrotic or inflammatory tissue rather than viable tumor. Prospective studies are warranted to determine whether ctDNA dynamics correlate more closely with survival outcomes than conventional RECIST-based radiographic response in tebentafusp-treated patients.

## Data Availability

The original data presented in the study are included in the article. Further inquiries can be directed to the corresponding author.
